# Breast Cancer Risk and Prevention: A Step Forward

**DOI:** 10.3390/cancers15235559

**Published:** 2023-11-23

**Authors:** Sofia Vidali, Tommaso Susini

**Affiliations:** 1 Breast Unit, Azienda Ospedaliera Universitaria Careggi, 50134 Florence, Italy; 2 Breast Unit, Gynecology Section, Department of Health Sciences, University of Florence, 50134 Florence, Italy

## 1. Introduction

Breast cancer (BC) is a leading topic in medical research as it is the most common cancer occurring in women worldwide; its incidence is progressively increasing in all age groups. However, its mortality is promisingly decreasing thanks to technological advances, therapy, and diagnostic improvements [1].

In an era of rapid scientific advancement, great efforts are being made to better understand and correct factors that may influence BC incidence and development and to further reduce mortality and morbidity rates by focusing research on new predisposing factors and improving both diagnostic and therapeutic approaches [2].

This Special Issue aims to present and explore, through the publication of 17 original articles, some of the emerging evidence, including specific molecular assets, life habits, predisposing breast lesions, as well as the issues of detection and therapeutic approaches. Thus, we aim to highlight the importance of a deeper and broader understanding that may allow for not only earlier and more efficient treatment but also effective primary prevention and incidence reduction in the most common cancer of the female population worldwide.

Although a comprehensive single model may still be unreliable due to lack of sufficient knowledge, single factor analysis and consequent risk stratification may be an appropriate step for actual disease management [3].

Predisposing factors can be divided into modifiable and non-modifiable. Among the latter, genetic assets have been observed to be directly or indirectly associated with BC, and some specific genetic mutations are known to greatly increase BC risk; the more that research advances, the more mutations and genetic variants that are found and defined [4].

Moving from the micro to macro level of non-modifiable risk factors, specific non-malignant breast lesions included in the B3 lesions of uncertain malignant potential represent quite a hot topic in breast disease management. In fact, although they are usually removed surgically, some international organizations and breast research guidelines suggest follow-up only or vacuum-assisted excision (VAE). The risk of upgrading to BC is known, but there are very few data focused on their role as a risk factor for future BC development and consequently of the need for surgical intervention rather than VAE or imaging follow-up [5].

As mentioned above, not to be forgotten are modifiable risk factors, which mainly include lifestyle and medications. In the work of Franchini et al., the weight on BC development risk of lifestyle asset was assessed through self-administered questionnaires from 5601 postmenopausal women then analyzed using a logistic model that combines a model-driven and data-driven approach to analysis: communities of women with common characteristics were identified and grouped into risk profiles using social network analysis techniques. The results suggested that prevention programs focused on increasing physical activity should be widely promoted, especially among the oldest women, while pregnancy, breastfeeding, salt restriction, and oral contraceptive (OC) use may have different effects on cancer risk depending on the woman’s overall risk profile [6].

In addition to the need for a deeper understanding of predisposing factors to directly influence the risk of developing BC, the continuous updating of and improvement in diagnostic and therapeutic strategies remain crucial and have not yet been established. In fact, diagnostic standards are not yet perfectly established, and screening programs, even in Western countries, are constantly being revised, with many efforts being made to improve them and implement them using the latest technologies [7,8].

The pattern of innovation also includes the improvement of well-established diagnostic and preventive models, which may help to identify standard pattern possible weaknesses and increase their effectiveness [9,10]. Leading in the research field is the automation of diagnostic imaging analysis and pathological feature identification: convolutional neural network (CNN) models have been proposed to automate breast density assessment, BC detection, and risk stratification [11].

Among the most known and debated of these possible weaknesses, one that was mostly investigated with already a solid base of evidence, is the role of additional non-mammographic imaging for screening programs, such as ultrasound (US) evaluation. In the work of Kuhl et al. on the J-START cohort, the utility of supplemental US in BC screening was investigated. This study found that supplemental US induced a reduction in interval cancer and late-stage disease rates, thus helping reduce BC-related mortality. Additionally, from a secondary analysis on breast density as a modulator of the results, they found that the contribution of ultrasonography to the early diagnosis of breast cancer in Japanese women did not depend on breast density. This is a striking result as the current evidence on US supplemental screening for BC in Western countries says that it is needed only for women with dense breasts [12,13].

Although the majority of average-risk screening studies compose their populations of women with dense breasts, a discrete number of others investigated the role of supplemental, non-mammographic imaging in BC diagnostic patterns irrespective of breast composition or enrolling patients with all grades of breast density and observed no differences in incidence based on supplemental imaging BC detection rates between dense and non-dense subgroups [14,15,16].

To conclude, a final tool to improve and become more effective in the management of BC is represented by the sharing of knowledge, both within the scientific community in order to uniform standards of care and to enrich each other’s results based on the union of data from heterogeneous populations and between the latter and patients. Their adherence to medical care and screening programs is necessary to improve diagnostic and therapeutic outcomes, and among the responsibilities of the scientific community, promoting patient health education must be a topic of primary interest.

## 2. An Overview of Published Articles

In the work of Akdeniz et al. (contributor 1), a sample of 1053 BC cases and 7094 controls from different regions of Norway were assessed for polygenic risk scores (PRSs), which showed an increased risk of BC in individuals in the highest decile of PRSs, which was double that of individuals with a median PRS.

In the paper by Verma et al. (contributor 2), who reviewed a genome-wide association study (GWAS) of breast density in 1323 women of African ancestry undergoing mammography, 34 significant loci associated with breast density, a known predisposing factor for BC, where identified, and the most significant of them showed a large overlap between previously identified breast cancer SNPs and SNPs associated with breast density.

As described by Berga-Švītiņa et al. (contributor 3) in their case–control genome-wide association study (GWAS), data of 406 germline BRCA1 pathogenic variants (PVs) (c.4035 del or c.5266dup) carriers affected by BC or ovarian cancer were compared to unaffected individuals; these pVs were shown to predict an individual’s risk of BC. 

In the work of Ho et al. (contributor 4) on 28,234 asymptomatic Asian women, where a Gail model was used to test the short- and long-term absolute risks of BC based on age at onset in different subgroups, they observed that family history of BC, positive recall status, and prior breast biopsy may lead to underestimation of risk, whereas being underweight is associated with overestimation of risk.

Peng et al. (contributor 5) investigated the interaction between the well-known advanced glycation end products (AGEs) and their receptor (RAGE), which induces oxidative stress and inflammation involved in the development of BC. They measured plasma levels in 1051 pairs of age-matched BC patients and controls and found that higher levels of AGEs and the AGEs/sRAGE ratio were associated with an increased risk of breast cancer, with a positive association between AGEs and poor prognosis, but sRAGE levels were negatively associated with breast cancer risk, especially in women <60 years of age.

Pistiolis et al. (contributor 6) focused on the antitumor effect of melatonin on BC, previously described in both in vitro and in vivo studies; however, melatonin was found to have a protective effect on BC-specific survival (BCSS) in univariate analysis in the 926 patients of their population who were prescribed with it, but when adjusting for known prognostic factors in multivariate analysis, this beneficial effect disappeared, making it impossible to show a strong association.

Song et al. (contributor 7) provide evidence of the impact of circadian disruptors (including night shift work, domestic light exposure at night, sleep duration, and circadian genes polymorphism) as risk factors for BC in a case–control study of 464 Chinese patient pairs, showing that the risk of BC was higher in the short sleep duration group, and that four SNPs in key circadian genes (CRY2 and PER1) were positively associated with breast cancer risk.

In the study by Bellini et al. (contributor 8) on the treatment and follow-up data of patients diagnosed with B3 lesions, an immediate upgrade of B3 lesions to BC was observed in 22.7% and at long-term follow-up of another 9.2%, supporting the idea that B3 lesions should be removed and suggesting annual screening mammography for women after a B3 diagnosis due to the significantly increased risk of B3⁵.

In the work of Franchini et al. (contributor 9), the weight on BC development risk of lifestyle asset was assessed through self-administered questionnaires from 5601 postmenopausal women; these were then analyzed using a logistic model that combines a model-driven and data-driven approach. Communities of women with common characteristics were identified and grouped into risk profiles using social network analysis techniques. The results suggested that prevention programs focused on increasing physical activity should be widely promoted, especially among the oldest women, while pregnancy, breastfeeding, salt restriction, and oral contraceptive (OC) use may have different effects on cancer risk depending on the woman’s overall risk profile.

The systematic review and meta-analysis by Barańska et al. (contributor 10) examined BC risk according to estrogen receptor (ER), progesterone receptor (PR), and human epidermal growth factor receptor 2 (HER2) status. The summary meta-analysis showed that the overuse of OCs was associated with a significantly increased risk of triple-negative BC and ER. There was also a significant reduction in the risk of ER+ BC and a small reduction in the risk of HER2+, suggesting that OC use has different effects on the risk of breast cancer subtypes defined by receptor status. 

In the work by Rigaud et al. (contributor 11), pre-trained EfficientNetB0 deep learning (DL) models for automated breast density assessment were used to retrospectively investigate 3052 women studied with different imaging tools (i.e., full-field digital mammogram [ffdm] and digital breast tomosynthesis [dbt]) and with at least three available exams with and without clinical information. This occurred in order to improve the reliability and versatility of the technique. Pre-trained EfficientNetB0 DL models with or without clinical history were optimized using BI-RADS versus binary (dense or non-dense) density classification, with the best breast density estimation achieved using full-field digital mammography and DBT images without added clinical information.

In the study by Li et al. (contributor 12), temporal analysis of annual screening mammograms using a long short-term memory (LSTM) network technique was evaluated to test its potential to identify women at risk of future BC. In this case–control work, biopsy-confirmed (malignant or benign) imaging abnormalities from a sample of women who also had previous imaging available were collected, and radiomic- and deep learning-based features were extracted on regions of interest and input into LSTM recurrent networks to classify whether the future lesion would be malignant or benign, using either all available previous time points or one single previous time point.

Classifiers incorporating multiple time points with LSTM, either based on deep learning-extracted features or radiomic features, were reported to perform better, whereas those using only a single time point failed to show improved performance compared to controls; similar results were observed when using features extracted from the affected versus contralateral breast in predicting future unilateral malignancy.

In the review of Lim et al. (contributor 13), incidence, mortality, early detection, mammography programs, and risk-based screening initiatives in screening programs in Asian countries are shown to vary from country to country, with some offering annual mammography from age 40, others biennial, and some other every three years. In addition, the mortality to incidence ratio, defined as the number of deaths compared to the number of BC diagnosed each year, a tool commonly used as a high-level comparative measure to identify inequalities in cancer outcomes, was observed to be higher than the world average in Asia and the second highest in the world by region.

Ding et al. (contributor 14) examined the short-term effectiveness of a mammography screening program for the major molecular subtypes of invasive BC, specifically the relationship between stage at diagnosis and method of detection (screen-detected or interval) and the relationship between method of detection and regularity of attendance (regular versus irregular) in different molecular subtypes of BC. Their results showed that screen-detected BC was more likely to be diagnosed at early stages than interval BC, and regular participation was associated with a higher likelihood of screening detection than irregular participation for luminal, luminal-HER2-positive, and triple-negative BC, making regular screening effective for all breast cancers except the HER2 subtype.

Ciabattoni et al. (contributor 15) in their work on the feasibility of Breast IRRADIATA (Italian Repository of RADIotherapy dATA), a collaborative nationwide project focused on BC patients treated with radiotherapy (RT) aimed to create a national registry and define the patterns of care in Italy, documented good feasibility of the questionnaires provided to participating centers. They demonstrated 100% response to all questions achieved with acceptable (10min) time for data entry/update, which may allow for a large application nationwide and a better assessment of the pattern of care.

As shown in the work of Nishimura et al. (contributor 16) where the impact of Breast Cancer Awareness Month (BCAM) on public awareness of this disease in the USA was analyzed using the relative search volume (RSV) of Google Trends, surrogate join points around BCAM for “breast cancer” every year from 2012 to 2021 were detected, with a significant increase in weekly RSVs. These results suggest that BCAM successfully improves public awareness and knowledge of the disease.

The work of Medina et al. (contributor 17) explored the impact of eHealth in overcoming barriers to access to conventional care in psychosocial interventions in BC-positive populations through the administration of newly diagnosed patients of ICOnnecta’t, a tiered digital ecosystem designed to build wellbeing and reduce psychosocial risks through screening and monitoring, psychoeducation campus, peer support community and online group psychotherapy. The results showed good user satisfaction, acceptance and use, and low attrition rates. A total of 443 patients’ needs were identified and responded to, resulting in 94.33% of users staying in the preventive steps without additional progression, with better psychosocial outcomes.

## 3. Conclusions

Although grouped in one single definition, breast cancer collects a discrete number of different pathological entities, each with their radiological, genetic, and prognostic characteristics; therefore, they are subjected to a wide range of predisposing conditions and are modifiable and non-modifiable. With the advance of technology, the aim of the scientific community must be to better define risk factors, from macroscopic to molecular and genetic ones, improve diagnostic and screening procedures, and share these advances both with colleagues and with patients in order to maximize survival and reduce mortality and incidence. The articles included in this Editorial are a valid example of the efforts made to improve, share, and step forward in order to manage women’s most frequently suffered disease.
